# Deep-learning image reconstruction for image quality evaluation and accurate bone mineral density measurement on quantitative CT: A phantom-patient study

**DOI:** 10.3389/fendo.2022.884306

**Published:** 2022-08-11

**Authors:** Yali Li, Yaojun Jiang, Xi Yu, Binbin Ren, Chunyu Wang, Sihui Chen, Duoshan Ma, Danyang Su, Huilong Liu, Xiangyang Ren, Xiaopeng Yang, Jianbo Gao, Yan Wu

**Affiliations:** Department of Radiology, The First Affiliated Hospital of Zhengzhou University, Zhengzhou, China

**Keywords:** bone mineral density, osteoporosis, deep learning iterative reconstruction, Catphan 500, European Spine Phantom

## Abstract

**Background and purpose:**

To investigate the image quality and accurate bone mineral density (BMD) on quantitative CT (QCT) for osteoporosis screening by deep-learning image reconstruction (DLIR) based on a multi-phantom and patient study.

**Materials and methods:**

High-contrast spatial resolution, low-contrast detectability, modulation function test (MTF), noise power spectrum (NPS), and image noise were evaluated for physical image quality on Caphan 500 phantom. Three calcium hydroxyapatite (HA) inserts were used for accurate BMD measurement on European Spine Phantom (ESP). CT images were reconstructed with filtered back projection (FBP), adaptive statistical iterative reconstruction-veo 50% (ASiR-V50%), and three levels of DLIR(L/M/H). Subjective evaluation of the image high-contrast spatial resolution and low-contrast detectability were compared visually by qualified radiologists, whilst the statistical difference in the objective evaluation of the image high-contrast spatial resolution and low-contrast detectability, image noise, and relative measurement error were compared using one-way analysis of variance (ANOVA). Cohen’s kappa coefficient (k) was performed to determine the interobserver agreement in qualitative evaluation between two radiologists.

**Results:**

Overall, for three levels of DLIR, 50% MTF was about 4.50 (lp/cm), better than FBP (4.12 lp/cm) and ASiR-V50% (4.00 lp/cm); the 2 mm low-contrast object was clearly resolved at a 0.5% contrast level, while 3mm at FBP and ASiR-V50%. As the strength level decreased and radiation dose increased, DLIR at three levels showed a higher NPS peak frequency and lower noise level, leading to leftward and rightward shifts, respectively. Measured L1, L2, and L3 were slightly lower than that of nominal HA inserts (44.8, 95.9, 194.9 versus 50.2, 100.6, 199.2mg/cm^3^) with a relative measurement error of 9.84%, 4.08%, and 2.60%. Coefficients of variance for the L1, L2, and L3 HA inserts were 1.51%, 1.41%, and 1.18%. DLIR-M and DLIR-H scored significantly better than ASiR-V50% in image noise (4.83 ± 0.34, 4.50 ± 0.50 versus 4.17 ± 0.37), image contrast (4.67 ± 0.73, 4.50 ± 0.70 versus 3.80 ± 0.99), small structure visibility (4.83 ± 0.70, 4.17 ± 0.73 versus 3.83 ± 1.05), image sharpness (3.83 ± 1.12, 3.53 ± 0.90 versus 3.27 ± 1.16), and artifacts (3.83 ± 0.90, 3.42 ± 0.37 versus 3.10 ± 0.83). The CT value, image noise, contrast noise ratio, and image artifacts in DLIR-M and DLIR-H outperformed ASiR-V50% and FBP (*P*<0.001), whilst it showed no statistically significant between DLIR-L and ASiR-V50% (*P*>0.05). The prevalence of osteoporosis was 74 (24.67%) in women and 49 (11.79%) in men, whilst the osteoporotic vertebral fracture rate was 26 (8.67%) in women and (5.29%) in men.

**Conclusion:**

Image quality with DLIR was high-qualified without affecting the accuracy of BMD measurement. It has a potential clinical utility in osteoporosis screening.

## 1 Introduction

The elderly men and postmenopausal women had a high incidence rate of osteoporosis and related vertebral fracture (1). Vertebral fracture, especially thoracolumbar osteoporotic compression fracture, often occurs in the mid-thoracic (T7-8) and thoracolumbar spine (T12-L1) (2, 3). Bone mineral density (BMD) obtained from quantitative computed tomography (QCT) is a volumetric measure of vertebral trabecular bone with high sensitivity and accuracy for predicting bone strength and fracture risk (4–6). QCT not only reduces the influence of overlying ribcage (2) but also prevents severe spinal degeneration and vascular calcification without requiring the oral contrast agent and body position (5) compared with dual-energy X-ray absorptiometry (DXA). QCT is superior to DXA in BMD measurement for early screening of osteoporosis. However, a high level of radiation exposure delivered to patients with QCT limits its further clinical application (6). Recently, the combination of low-dose CT (LDCT) and lumbar QCT has been initiated by the China Health Big Data (China Biobank) project for opportunistic screening of osteoporosis and lung cancer simultaneously in terms of reducing radiation dose, repeated scan, patient time, and additional costs. Wu et al. (5) described the study protocol of the combination of QCT with LDCT. Inherently, Cheng et al. (7) conducted a multicenter population-based cohort study with QCT to determine the prevalence of osteoporosis in China.

Unfortunately, image noise increased obviously after reducing radiation dose, while image quality decreased significantly, particularly in the spine (5), contributing to an inevitable decrease in diagnostic performance. An iterative reconstruction (IR) algorithm is introduced to reduce image noise and preserve image quality between radiation risk and diagnostic performance (8, 9). But many IR algorithms can change the magnitude of the image noise and texture details and may cause an adverse impact on the detection of low-contrast lesions, particularly at high strength levels (10–12).

Currently, a new-generation deep-learning image reconstruction (DLIR) (TrueFidelity, GE Healthcare) was proposed to improve the CT image quality. It utilizes deep neural networks that consist of layers of mathematical equations, with millions of connections and parameters to generate CT images, and is designed with a fast reconstruction speed for routine CT use, even in acute care settings. And it consists of three selectable reconstruction strength levels (low, medium, and high) to control the amount of noise reduction corresponding to clinical applications and radiologist preference (13).

To assess the image quality of LDCT, accurate BMD measurement, and the performance of DLIR for image quality at ultralow-dose level, Li et al. (14) systemically evaluated the physical image quality on Catphan 500 phantom. Results indicated that the CT number linearity was unbiasedly contributing to accurate BMD quantification. DLIR performed better than iterative model reconstruction (IMR, level 2) at 0.25 and 0.75 mGy, but they didn’t evaluate the accuracy of BMD value on European Spine Phantom (ESP). Therefore, on the basis of Li et al.’s experiment, our study aimed to evaluate CT image quality and accurate BMD measurement on the Catphan 500 phantom and ESP and patient study using DLIR algorithm in comparison to 50% adaptive statistical iterative reconstruction-veo (ASiR-V 50%) and filtered back projection (FBP) reconstruction algorithms.

## 2 Materials and methods

This prospective study was strictly adhered to HIPAA Privacy Rule and approved by the ethics committee of the First Affiliated Hospital of Zhengzhou University and Beijing Jishuitan Hospital. The China Biobank project is a multicenter cohort study and has been registered with the US clinical trials database (https://clinicaltrials.gov/ct2/show/NCT03699228; trial identifier: NCT03699228). Our hospital is one of the collaborating hospitals and provided the patient cohort for this study. The informed consent of the patients was all obtained.

Data acquisitions were obtained from Catphan 500 phantom (Phantom Laboratory, Salem, NY, USA) and ESP (No. 145, Germany ORM company), as well as patients on Revolution CT (GE Healthcare, WI, USA) from April 2020 to June 2021. The weekly air calibration and monthly QA were performed by qualified technologists before data acquisitions and BMD measurement throughout the whole study using the Model 3 synchronous QA phantom. To reduce the uncertainty of measurements, data acquisitions were scanned 10 consecutive times separately on Catphan 500 and ESP without repositioning.

### 2.1 Catphan 500 Phantom

The Catphan 500 phantom consists of 4 modules, including CTP401, CTP528, CTP515, and CTP486 modules. The module CTP528, CTP515, and CTP486 were selected to evaluate the high-contrast spatial resolution, low-contrast detectability, and image noise, respectively (15).

### 2.2 European Spine Phantom

ESP consisted of water-equivalent plastic made of epoxy resin and 3 cylindrical inserts of artificial vertebrae with nominal trabecular BMD values of L1 (50.5mg/cm^3^), L2 (100.6mg/cm^3^), and L3 (199.2mg/cm^3^), which are equivalent to water and bone solid compartments that simulate lumbar spine of the human body (16).

### 2.3 Study participants

A total of 716 patients (300 women and 416 men, age, 62.4 ± 7.2 years, range, 55-78 years) who derived from the China Biobank Study were prospectively enrolled in our hospital during March and June 2021 (**Table 1**). The exclusion criteria included: patients aged below 50 years old; patients with the use of oral corticosteroids or anti-osteoporotic medication such as vitamin D supplementation; and patients with metal implants in the upper abdominal.

**Table 1:** Summary of data acquisitions at two phantoms and clinical setting of patient.

CT parameters	Catphan 500	ESP	Participants
Acquisition mode	Axial/Helical	Axial/Helical	Helical
Reconstruction kernel	Standard	Standard	Standard
Tube voltage (kV)	120	120	120
Tube current-time product (mAs)	25/75	25/75	25/75
Thickness/increment (mm)	1.25/5	1.25/5	1.25/5
Pitch	0.992	0.992	0.992
Beam collimation (mm)	40	40	40
DFOV (mm)	500	500	500
Matrix size	512×512	512×512	512×512
X-ray tube rotation speed(s/r)	0.5	0.5	0.5
Reconstruction algorithm	FBP/ASiR-V50%/DLIR(L/M/H)	FBP/ASiR-V50%/DLIR(L/M/H)	FBP/ASiR-V50%/DLIR(L/M/H)
Detector configuration (mm)	256×0.625	256×0.625	256×0.625
Voxel size (mm)	0.61	0.61	0.61
CTDI_vol_ (mGy)	0.25/0.75	0.25/0.75	0.25/0.75

CT, computed tomography; ESP, European Spine Phantom; FBP, filtered back projection; ASiR-V50%, adaptive statistical iterative reconstruction-veo 50%; DLIR(L/M/H), deep-learning image reconstruction, level low, medium, and high; CTDI_vol_, volume CT dose index; mGy, milligray; DFOV, display field of view.

### 2.4 Scan protocol

Data acquisitions were obtained with a fixed tube voltage of 120 kV. And the tube current was set to yield a volume CT dose index (CDTI_vol_) at 2 ultralow-dose levels of 0.25 and 0.75mGy. Images were reconstructed using FBP, ASiR-V50% and DLIR (level, low, medium, and high) with a standard kernel (**Table 1**).

### 2.5 Data measurement and image evaluation

High-contrast spatial resolution, low-contrast detectability, and image noise are the standard image quality parameters of CT system.

#### 2.5.1 High-contrast spatial resolution

High-contrast spatial resolution indicates the capability of a CT system to differentiate the small high-contrast objects (15). The module CTP528 is used to measure the high-contrast spatial resolution *via* subjective and objective evaluation. For subjective evaluation, two radiologists with 6 and 8 years of radiological experience visually assess the 21 lp/cm high-resolution gauges by adjusting the window width (WW) and window level (WL) until resolving the highest number of visible line pairs. For objective evaluation, the MTF curve that represents the imaging capability of CT system for different frequency components is used to distinguish the line pairs to decimal level, and analyze the curve trend in the low- and high-frequency ranges (15).

#### 2.5.2 Low-contrast detectability

Low-contrast detectability determines the capability to distinguish different lesions with a minor density difference (17). The The module CTP515 consists of 3 groups supra-slice targets at the contrast levels of 1%, 0.5%, and 0.3% with the diameter of 15, 9, 8, 7, 6, 5, 4, 3, and 2 mm, respectively. The low-contrast detectability is estimated by the nominal contrast level of 1.0% (15). Two radiologists independently and blindly adjusted the WW and WL to identify the smallest supra-slice target diameter and performed a direct side-by-side comparison (18).

#### 2.5.3 Image noise

Image noise represents the standard deviation of CT values within an ROI in the uniform phantom image (15). The noise power spectrum (NPS) is used to calculate the noise characterization, and the NPS curve reflects the variation of image intensity over high-contrast resolution frequency (19). The CTP489 module is an image uniformity module that is cast from uniform material with the CT number within 2% of water density (-25~25HU). Five circular regions of interest (ROIs) with radii of 5-6mm were cropped in the central and peripheral sites of the image (clock positions 12, 3, 6, and 9). The image uniformity was measured by the deviation of the minimum and maximum CT number values between central and peripheral sites and recommended within ±4HU (15, 20).

#### 2.5.4 Bone mineral density measurement

CT images were transferred to a dedicated QCT PRO BMD workstation (Mindways QCT PRO workstation). All QCT analyses were performed by professionally trained radiologists using Mindways QCT PRO software (3D spine function version 6.10, Mindways software Inc., Austin, TX, USA) and conducted by a Mindways QCT-PRP operator’s manual (21).

Firstly, start the QCT PRO software, click on the 3D Spine Analysis module button, and select the L1, L2, and L3 HA inserts to analyze. Then, click the rotation tab, drag the yellow crosshair to the center of L1, L2, and L3 on the sagittal image, rotate them until it resembles a vertical box, mark the middle of them on the coronal images, and correlate to the corresponding axial images. Finally, set 3 ROIs at L1, L2, and L3 with the circular area of about 2/3 in the entire axial image and slice thickness of 9 mm, click the report tab, and calculate the BMD of L1, L2, and L3. Unless obvious errors occurred in the measurement process, workstation software were processed for automatic analysis, including automatic functions, automatic detection of boundaries, and automatic generation of ROIs throughout the whole operation.

#### 2.5.5 Accurate bone mineral density quantification

The accuracy of the BMD value on QCT is evaluated by calculating the measurement error for each HA insert. Measurement error is defined as a deviation between the measured HA and true HA concentration (units: mg/cm^3^). Relative measurement error reflects the accuracy error in proportion to true HA concentration (16, 22). The precision error is used to interpret significant changes in BMD and expressed as the percentage coefficient of variation (%CV) (23).


(1) $$=\frac{Measurement\;error(mg/cm^{3})}{\text{Measured HA concentration-true HA concentration}}$$

(2) $$\text{Relative measurement }error(\%)=\frac{Measurement\;error(mg/cm^{3})}{True\;HA\;concentration(mg/cm^{3})}\times 100$$

(3) $$\%CV=\frac{SD}{Mean}\times 100$$

#### 2.5.6 Qualitative image analysis

Two radiologists independently and blindly assess the image quality of CT images using a point-based Likert scale (**Table 2**) (19). Patient information and examination details were anonymized, images were presented in a random order, and radiologists were allowed to freely scroll or zoom the images and adjust the WW/WL. Consensus reading was used when there was any disagreement between two radiologists.

**Table 2:** Grading scale of the qualitative image analysis.

Grading score	Image noise	Image contrast	Small structure visibility	Image sharpness	Artifacts
1	Unacceptable	Unacceptable	Unacceptable	Severe	Severe
2	Above average	Suboptimal	Suboptimal	Moderate	Major
3	Average	Acceptable	Acceptable	Minimal	Minor
4	Less than average	Above average	Above average	No blurring	None
5	Minimal	Excellent	Excellent		

#### 2.5.7 Quantitative image analysis

The circular ROIs with radii of 7 mm were manually drawn on the lung, air, liver parenchyma, and right side of the paraspinal muscle in five image sets to measure the mean CT value and SD in Hounsfield units (HU).

Lung measurements were obtained from the lower lung lobes toward the periphery, liver measurements from the liver parenchyma avoiding large vessels and biliary tree, air measurements were defined as the SD of air external and anterior to the patient at the sternomanubrial junction, and muscle measurements were measured at the right side of the paraspinal muscle of the posterior margin of the L2 vertebra. The SD of air and muscle were considered as image noise for chest and abdomen (8, 24).


(4) $$N\text{oise}=SD_{background}$$

(5) $$CNR=\frac{ROI_{organ}-ROI_{\text{background}}}{SD_{\text{background}}}$$

where *ROI_organ_* and *ROI_background_* refer to the mean CT value of the lung, liver parenchyma, air, and paraspinal muscle, respectively; *SD_organ_* and *SD_background_* are image noise determined as SD in the lung, liver parenchyma, air, and muscle, respectively.

### 2.6 Statistical analysis

All statistical analyses were performed using SPSS 20 software (IBM Corp., Armonk, NY, USA). The MTF and NPS curves were calculated with MATLAB R2018b (MathWorks, Natick, MA, USA). The continuous variables were expressed as mean ± SD. Subjective evaluation of the image high-contrast spatial resolution and low-contrast detectability were compared visually by qualified radiologists, whilst the statistical difference of objective evaluation of the image high-contrast spatial resolution, low-contrast detectability, image noise, and relative measurement error were compared using one-way analysis of variance (ANOVA) and Bonferroni correction. Friedman test was used to perform the qualitative evaluation. Cohen’s kappa coefficient (k) was used to determine the interobserver agreement between two radiologists. A Kappa value of 0.21-0.40 was defined as poor, 0.41-0.60 as moderate, 0.61-0.80 as substantial, and 0.81-1.00 as excellent. A *P*<0.05 was considered as statistically significant.

## 3 Results

### 3.1 High-contrast spatial resolution

#### 3.1.1 Subjective evaluation

**Figure 1:**
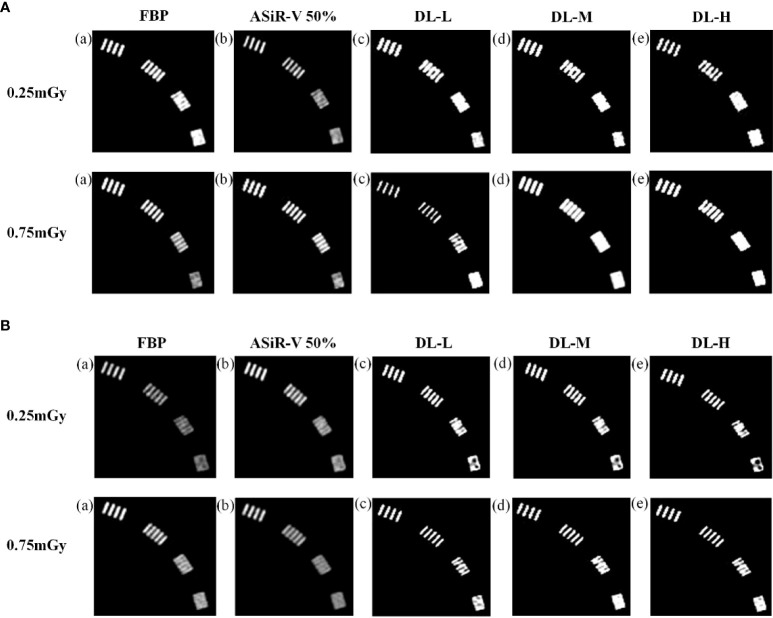
High-contrast images in helical mode reconstructed with FBP (a, f), ASiR-50% (b, g), and DLIR (L/M/H) (c, h; d, i; e, j) at 0.25mGy and 0.75mGy with a slice thickness of 1.25mm **(A)** and 5mm **(B)**, respectively. CT, computed tomography; FBP, filtered back projection; ASiR-50%, adaptive statistical iterative reconstruction-veo 50%; DLIR(L/M/H), deep-learning image reconstruction, level low, medium, and high; mGy, milligray.

**Figure 2:**
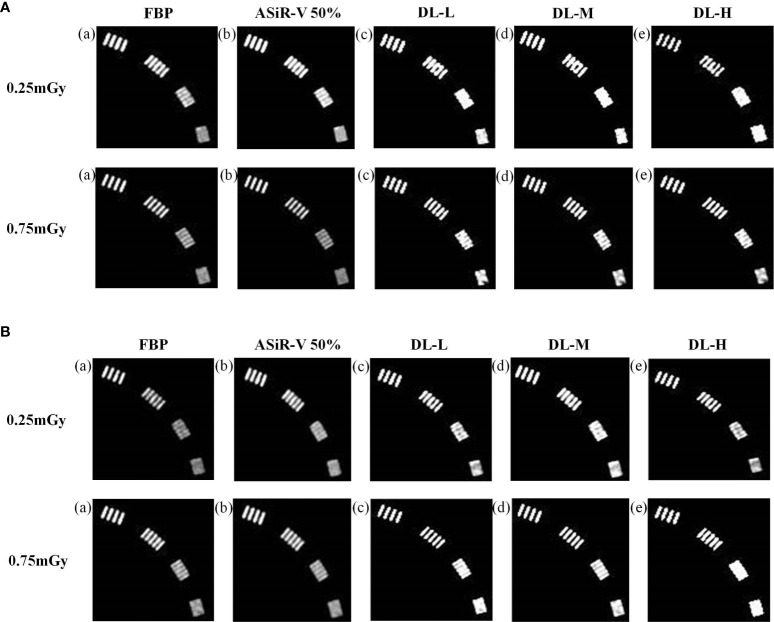
High-contrast images in axial mode reconstructed with FBP (a, f), ASiR-50% (b, g), and DLIR at three levels (L/M/H) (c, h; d, i; e, j) at 0.25mGy and 0.75mGy with a slice thickness of 1.25mm **(A)** and 5mm **(B)**, respectively. CT, computed tomography; DLIR(L/M/H), deep-learning image reconstruction, level low, medium, and high; mGy, milligray; ASiR-50%, adaptive statistical iterative reconstruction-veo 50%.

In general, the high-resolution bars were clearly separable at 6 lp/cm, but started blurring at 7 or 8 lp/cm, the resolving power was all high-qualified (**Figures 1**, **2**). The bars of the three levels of DLIR at 0.25mGy were comparable to those of ASiR-V50% at 0.75mGy. There were no statistically significant differences in slice thickness and scan type (*P*>0.05).

#### 3.1.2 Objective evaluation

**Figure 3:**
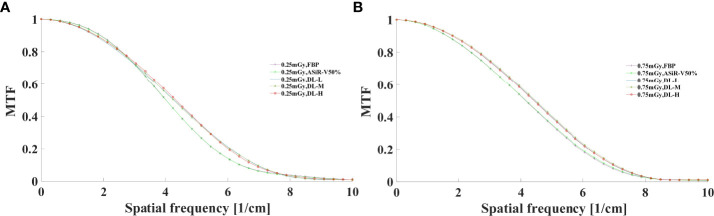
MTF curves in helical mode reconstructed with FBP, ASiR-V50%, and DLIR (L/M/H) at 0.25mGy **(A)** and 0.75mGy **(B)**. CT, computed tomography; FBP, filtered back projection; ASiR-50%, adaptive statistical iterative reconstruction-veo 50%; DLIR(L/M/H), deep-learning image reconstruction, level low, medium, and high; mGy, milligray.

**Figure 4:**
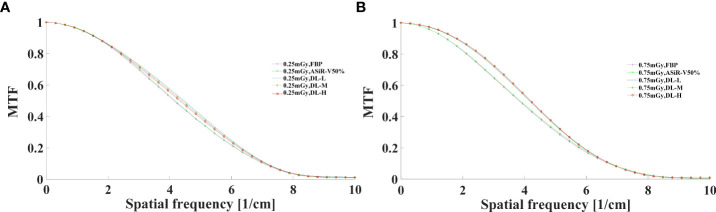
MTF curves in axial mode reconstructed with FBP, ASiR-50%, and DLIR (L/M/H) at 0.25mGy **(A)** and 0.75mGy **(B)**. CT, computed tomography; DLIR(L/M/H), deep-learning image reconstruction, level low, medium, and high; mGy, milligray; ASiR-50%, adaptive statistical iterative reconstruction-veo 50%.

The MTF values of FBP and ASiR-V50% at 50%MTF were ≤ 4.00lp/cm or less, while that of DLIR at three levels was at 4.50lp/cm. The resolving power at 10%MTF (6.78 ± 0.40 lp/cm) was generally similar to the subjective evaluation results, which showed no significant difference from that at 5%MTF. Thus, it could be used to evaluate the high-contrast spatial resolution of the CT system (**Figures 3**, **4**). The differences were not significant in slice thickness and scan type (*P*>0.05). The MTF value of DLIR (three levels) at 0.25mGy was comparative to that of FBP but slightly better than that of ASiR-V50% at 0.75mGy.

### 3.2 Low-contrast detectability

**Figure 5:**
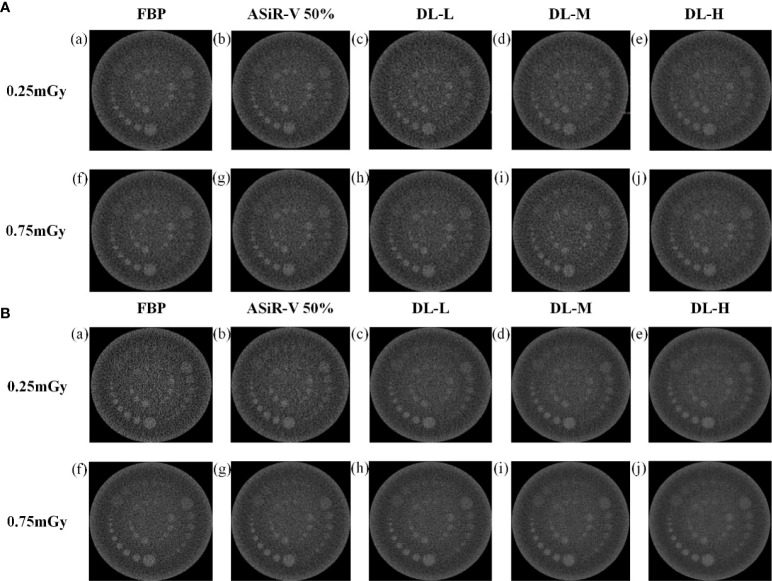
Low-contrast detectability images in helical mode reconstructed with FBP (a, f), ASiR-50% (b, g), and DLIR(L/M/H) (c, h; d, i; e, j) at 0.25mGy and 0.75mGy with a slice thickness of 1.25mm **(A)** and 5mm **(B)**, respectively. CT, computed tomography; FBP, filtered back projection; ASiR-50%, adaptive statistical iterative reconstruction-veo 50%; DLIR(L/M/H), deep-learning image reconstruction, level low, medium, and high; mGy, milligray.

**Figure 6:**
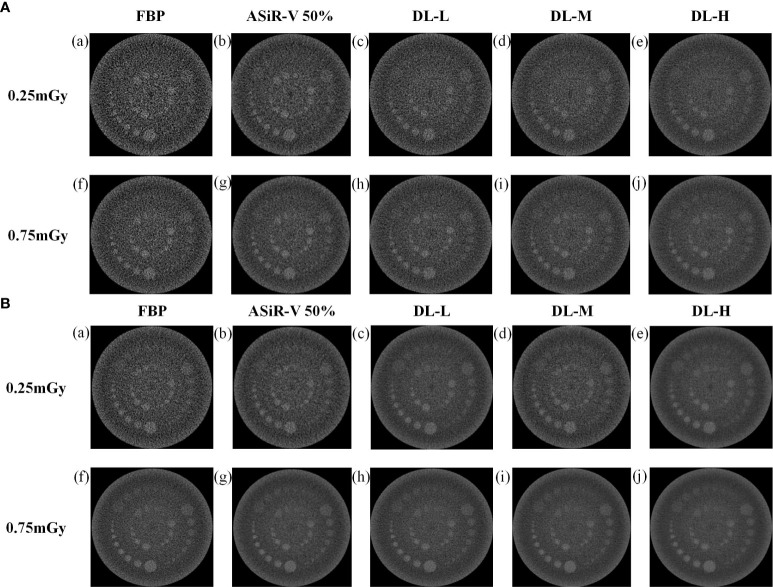
Low-contrast detectability images in axial mode reconstructed with FBP (a, f), ASiR-50% (b, g), and DLIR(L/M/H) (c, h; d, i; e, j) at 0.25mGy and 0.75mGy with a slice thickness of 1.25mm **(A)** and 5mm **(B)**, respectively. CT, computed tomography; DLIR(L/M/H), deep-learning image reconstruction, level low, medium, and high; mGy, milligray; ASiR-50%, adaptive statistical iterative reconstruction-veo 50%.

All CT images were visualized at a fixed window setting (WW/WL, 70/100 HU) (**Figures 5**, **6**). In general, the 3 mm low-contrast object at a 0.5% contrast level was clearly resolved, the 2 mm low-contrast object could be resolved for DLIR at three levels, and the diameters were all less than 5mm, which confirmed that the images were qualified (25). In respect of low-contrast detectability, DLIR-M and DLIR-H were superior to ASiR-V50%, DLIR-L was comparable to ASiR-V50% and better than FBP, and DLIR (three levels) at 0.25mGy was comparable to ASiR-V50% at 0.75mGy. Although DLIR were clearer as the strength level, slice thickness, and radiation dose increased, there was a slightly significant difference in scan type (*P*>0.05).

### 3.3 Image noise

**Figure 7:**
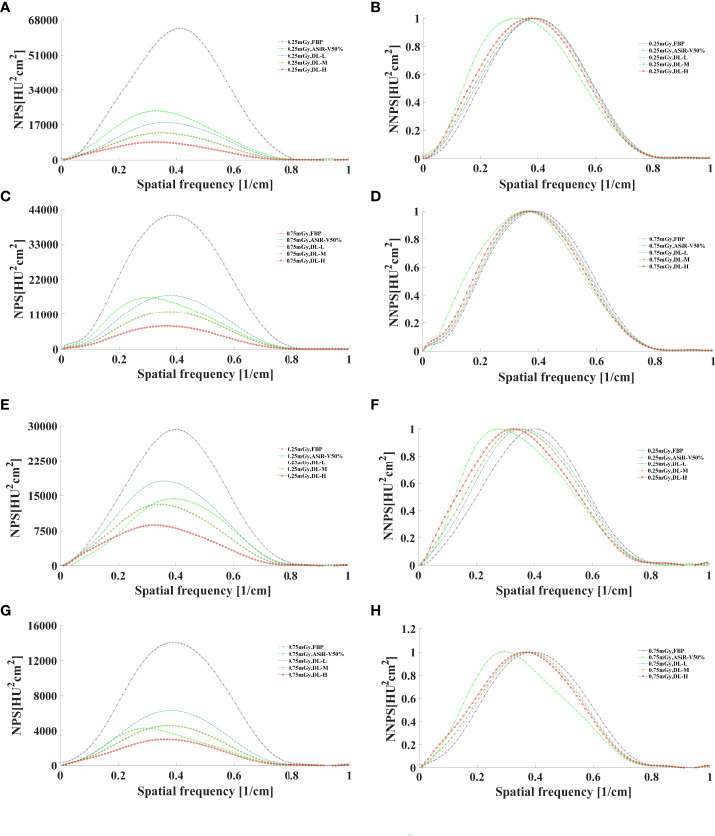
The curves of NPS and NNPS in helical mode reconstructed with FBP, ASiR-V50%, and DLIR (L/M/H) at 0.25mGy **(A, B, E, F)** and 0.75mGy **(C, D, G, H)** with a slice thickness of 1.25mm **(A–D)** and 5mm **(E–H)**. NPS, noise power spectrum; NNPS, normalized noise power spectrum; HU, Hounsfield units; FBP, filtered back projection; ASiR-50%, adaptive statistical iterative reconstruction-veo 50%; DLIR(L/M/H), deep-learning image reconstruction, level low, medium, and high; mGy, milligray.

**Figure 8:**
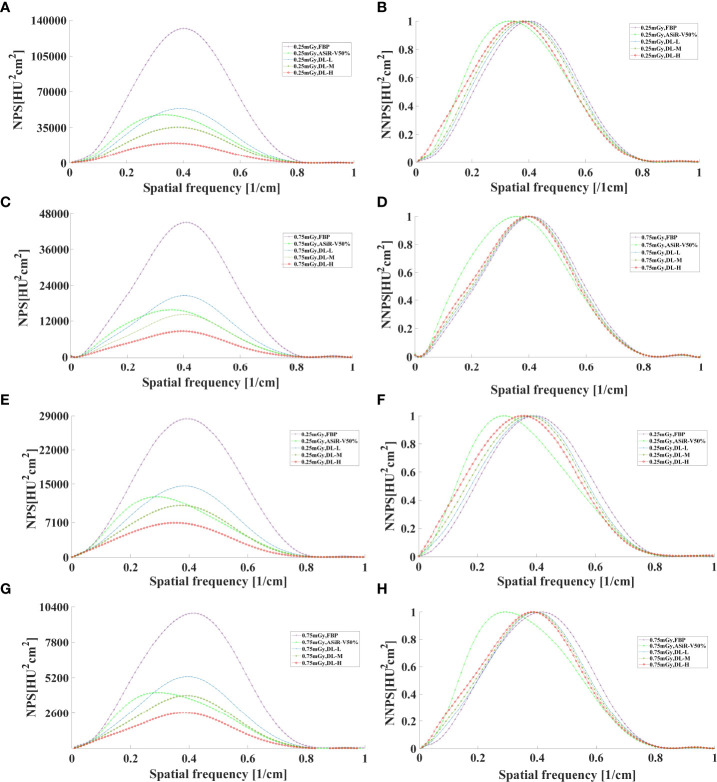
The curves of NPS and NNPS in axial mode reconstructed with FBP, ASiR-V50%, and DLIR (L/M/H) at 0.25mGy **(A, B, E, F)** and 0.75mGy **(C, D, G, H) **with a slice thickness of 1.25mm **(A–D)** and 5mm **(E–H)**. NPS, noise power spectrum; NNPS, normalized noise power spectrum; HU, Hounsfield units; FBP, filtered back projection; ASiR-50%, adaptive statistical iterative reconstruction-veo 50%; DLIR(L/M/H), deep-learning image reconstruction, level low, medium, and high; mGy, milligray.

In general, as the strength level decreased and the radiation dose increased, the noise level decreased while the peak frequency of the NPS curve increased (**Figures 7**, **8**). DLIR-M and DLIR-H achieved a lower noise level than FBP and ASiR-V50%, whilst DLIR-L was comparative to ASiR-V50%. The peak frequency of the NPS curve was higher at 0.75mGy than at 0.25mGy, and those of DLIR (three levels) at 0.25mGy and ASiR-V50% at 0.75mGy were comparable. Increasing the radiation dose, the NNPS curve of FBP and ASiR-V50% indicated a rightward in the peak frequency. As the strength level increased and radiation dose decreased, the NNPS curve of DLIR at three levels presented a leftward shift in the peak frequency and showed a similar shape with only a slight frequency shift under all scan protocols (**Figures 7**, **8**).

### 3.4 Accuracy of bone mineral density

**Figure 9:**
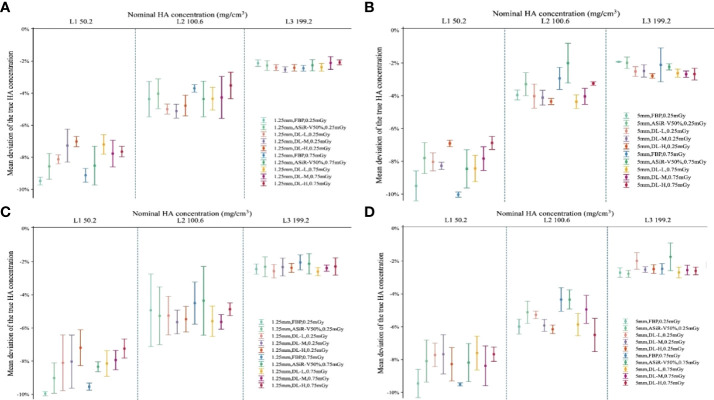
Accuracy deviation of bone mineral density in L1, L2, and L3 with ESP. Error bars standard deviation indicated the relative accuracy error (%) of 3 nominal HA concentrations (ESP, No.145; L1, 50.2; L2, 100.6; L3, 199.2 mg/cm^3^ HA) for helical **(A, B)** and axial **(C, D)** scan type. The relative measurement errors and coefficient of variation of L1, L2, and L3 were fell within the range of 4-15%, indicating no statistically significant differences among L1, L2, and L3 at different scan protocols (*P*>0.05). ESP, European Spine Phantom; HA, calcium hydroxyapatite; FBP, filtered back projection; ASiR-V50%, adaptive statistical iterative reconstruction-veo 50%; DLIR(L/M/H), deep-learning image reconstruction, level low, medium, and high; mGy, milligray.

Measured BMD of L1, L2, and L3 was slightly lower than that of nominal HA inserts (45.8, 95.9, 194.9 versus 50.2, 100.6, 199.2mg/cm^3^, respectively). The measurement error for L1, L2, and L3 HA inserts was 4.9, 4.1, and 5.1mg/cm^3^, with a relative measurement error of 9.84%, 4.08%, and 2.60%, respectively. Coefficients of variance for the L1, L2, and L3 HA inserts were 1.51%, 1.41%, and 1.18%. There were no statistically significant differences among L1, L2, and L3 under all scan protocols (*P*>0.05). The accuracy of BMD value varied greatly with FBP but little with DLIR in L1, L2, and L3, and BMD in L1 varied mostly compared with L2 and L3 (**Figure 9**).

### 3.5 Basic characteristics with participants

Of the 716 patients including 300 women and 416 men, with an age of 62.40 ± 7.20 (50-97) years, a body weight 63.07 ± 10.82 (45.00-76.50) kg, a height of 1.66 ± 0.69 (1.55-1.78) m, and BMI of 23.05 ± 3.58 (16.65-26.93) kg/m^2^ were recruited. The prevalence of osteoporosis was found in 74 (24.67%) women and 49 (11.79%) men, while osteoporotic vertebral fracture rate was observed in 26 (8.67%) women and 22 (5.29%) men (**Table 3**).

**Table 3:** Demographic characteristics of patient study.

Basic characteristics	Female patients (n=300)	Male patients (n=416)	Total patients (n=716)
**Age (years)**	58.86 ± 6.90 (range, 50-97)	63.86 ± 8.03 (range, 52-89)	62.40 ± 7.20 (range, 50-97)
**Weight (kg)**	57.29 ± 9.57	67.29 ± 8.19	62.29 ± 10.22
**Height (m)**	1.61 ± 0.06	1.70 ± 0.05	1.68 ± 0.07
**BMI (kg/m^2^)**	22.06 ± 3.43	23.39 ± 3.17	23.05 ± 3.58
**BMD (mg/cm^3^)**	63.96 ± 28.75	82.51 ± 47.30	73.24 ± 40.22
**Osteoporosis n [%]**	74 (24.67%)	49 (11.79%)	123 (17.18%)
**Vertebral fracture n [%]**	26 (8.67%)	22 (5.29%)	48 (6.70%)

Continuous variables are expressed as mean± standard deviation unless otherwise indicated. BMI, body mass index; BMD, bone mineral density.

### 3.6 Qualitative image analysis

**Figure 10:**
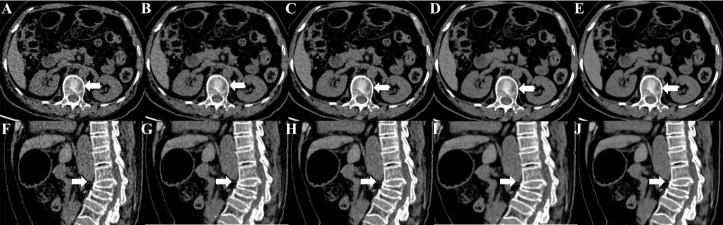
Unenhanced CT images of a 67-year-old female for osteoporotic vertebral fracture in the L3 vertebrae. CT images were reconstructed with FBP **(A, F)**, ASiR-V50% **(B, G)**, DLIR-L **(C, H)**, DLIR-M **(D, I)** and DLIR-H **(E, J)** with a slice thickness of 1.25mm at 0.75 mGy. The L3 vertebrae body was shown as a severe collapse in sagittal images (arrow), and the vertebral compression appearance was presented in axial images (arrow). The BMD values of FBP, ASiR-V50%, DLIR-L, DLIR-M and DLIR-H were 72.49, 72.74, 71.68, 70.11 and 69.24 mg/cm^3^ for L1 vertebrae, 67.33, 69.11, 70.25, 65.38, 68.49 mg/cm^3^ for L2 vertebrae, 62.08, 45.92, 49.57, 52.21, 50.93mg/cm^3^ for L3 vertebrae, respectively. CT, computed tomography; FBP, filtered back projection; ASiR-V50%, adaptive statistical iterative reconstruction-veo 50%; DLIR(L/M/H), deep-learning image reconstruction, level low, medium, and high; mGy, milligray; BMD, bone mineral density.

DLIR-M and DLIR-H were scored significantly better than ASiR-V50% in image noise (4.83 ± 0.34, 4.50 ± 0.50 vs 4.17 ± 0.37), image contrast (4.67 ± 0.73, 4.50 ± 0.70 vs 3.80 ± 0.99), small structure visibility (4.83 ± 0.70, 4.17 ± 0.73 vs 3.83 ± 1.05), image sharpness (3.83 ± 1.12, 3.53 ± 0.90 vs 3.27 ± 1.16), and artifacts (3.83 ± 0.90, 3.42 ± 0.37 vs 3.10 ± 0.83). There were statistically significant differences among DLIR-L, DLIR-M, and DLIR-H in all image quality metrics (*P*<0.001) (**Figure 10** and **Table 4**). The interobserver agreement between two radiologists showed an excellent agreement with a kappa value of 0.852.

**Table 4:** The qualitative image analysis.

Variables	FBP	ASiR-V50%	DLIR-L	DLIR-M	DLIR-H	*P*
Image noise	3.83 ± 0.37	4.17 ± 0.37	4.23 ± 0.31	4.50 ± 0.50	4.83 ± 0.34	<0.001
Image contrast	3.33 ± 1.25	3.80 ± 0.99	4.00 ± 0.35	4.50 ± 0.70	4.67 ± 0.73	<0.001
Small structure visibility	3.50 ± 1.31	3.83 ± 1.05	4.01 ± 0.53	4.17 ± 0.73	4.83 ± 0.70	<0.001
Image sharpness	2.17 ± 1.16	3.27 ± 1.16	3.22 ± 0.70	3.53 ± 0.90	3.83 ± 1.12	<0.001
Artifacts	2.81 ± 1.18	3.10 ± 0.83	3.17 ± 0.53	3.42 ± 0.37	3.83 ± 0.90	<0.001

FBP, filtered back projection; ASiR-V50%, adaptive statistical iterative reconstruction-veo 50%; DLIR(L/M/H), deep-learning image reconstruction, level low, medium, and high; There showed significant statistical differences across 3 levels of DLIR (P<0.001).

### 3.7 Quantitative image analysis

The overall image quality, CT value, image noise, CNR, and image artifacts were outperformed for DLIR compared with ASiR-V50% and FBP (*P*<0.001), whilst it was not a statistically significant difference between DLIR-L and ASiR-V50% (*P*>0.05). As radiation dose and strength level increased, image noise significantly decreased, CNR obviously increased, whilst CT value showed no significant difference (**Table 5**).

**Table 5:** Quantitative image analysis in patient study.

Variables	FBP	ASiR-V50%	DLIR-L	DLIR-M	DLIR-H	*P*
The mean CT value (HU)						
Lung	37.77 ± 2.82	40.57 ± 2.04	37.97 ± 3.32	38.10 ± 3.25	38.20 ± 3.40	0.875
Air	-872.87 ± 18.26	-872.53 ± 18.57	-873.67 ± 18.75	-872.77 ± 19.14	-871.63 ± 18.88	1.000
Liver	65.17 ± 3.07	65.77 ± 2.83	65.93 ± 4.00	66.00 ± 4.38	65.87 ± 4.77	0.999
Muscle	52.87 ± 2.50	53.90 ± 2.25	53.33 ± 2.75	52.90 ± 2.97	52.43 ± 3.21	0.986
Image noise (HU)						
Lung	15.60 ± 1.40	10.50 ± 1.90	9.60 ± 0.20	7.40 ± 0.10	5.35 ± 0.55	0.002^*^
Air	48.80 ± 0.00	43.25 ± 0.55	38.90 ± 0.70	34.30 ± 0.90	31.10 ± 1.00	<0.001^*^
Liver	19.35 ± 1.85	19.05 ± 2.65	13.30 ± 0.90	10.10 ± 0.90	7.40 ± 0.30	0.002^*^
Muscle	19.75 ± 1.15	15.10 ± 0.50	11.15 ± 0.75	8.45 ± 0.35	6.15 ± 0.05	<0.001^*^
CNR						
Lung	20.18 ± 0.28	21.55 ± 0.14	19.58 ± 0.01	21.50 ± 0.34	21.69 ± 0.22	<0.001^*^
Liver	0.67 ± 0.01	1.12 ± 0.08	1.16 ± 0.03	1.45 ± 0.05	2.23 ± 0.07	<0.001^*^

Data is expressed as mean ± standard deviation (SD); ^*^P<0.05; mGy, milligray; HU, Hounsfield units; FBP, filtered back projection; ASiR-V50%, adaptive statistical iterative reconstruction-veo 50%; DLIR(L/M/H), deep-learning image reconstruction, level low, medium, and high; CNR, contrast-to-noise ratio.

## 4 Discussion

In our study, we systematically evaluated the image quality, accurate BMD measurement, and clinical applicability of QCT with DLIR based on multi-phantom and patient studies. Results indicated great clinical importance without requiring any additional equipment and patient time, repeated CT scan, radiation dose, and additional costs. To our knowledge, it is the first systemic study to research the application of BMD measurements at an ultralow-dose level. QCT can be utilized for further opportunistic screening of osteoporosis, osteoporotic fracture, or other clinical applications (e.g., health check-ups) in China or worldwide countries accessing to CT easily than DXA (7).

Our results are consistent with Li et al. (15) findings on Catphan 500. For three levels of DLIR, MTF value at 50%MTF was about 4.50lp/cm, better than those for FBP (4.12 lp/cm) and ASiR-V50% (4.00 lp/cm). The 2 or 3 mm low-contrast object was clearly resolved at a 0.5% contrast level or at FBP and ASiR-V50%. Abdullah et al. (16) reported that the 50%MTF value and smallest size of objects were about 0.41 lp/cm and 3mm with ASiR-V (level: 40% and 60%), slightly lower than 4.50lp/cm and 2mm with DLIR. It showed an obviously lower NPS peak frequency and noise level, and a shift towards a lower spatial frequency in NNPS curve. As the strength level increased, the peak and spatial frequency of NPS curves with DLIR were decreased, which is consistent with a study reported by Greffier et al. (26). DLIR has been developed to reduce radiation dose and maintain image quality without changing the image texture or affecting the anatomical and pathological structures (13). And it can decrease the low-frequency noise component to improve low-contrast detectability for soft tissues ranging from 50 to 200 HU in abdominal CT (27), while maintaining the high-contrast spatial resolution of detailed structures, such as sharp edges and vessel boundaries at a low-dose level.

For image analysis in patients, DLIR-M and DLIR-H were scored better than ASiR-V50% in image noise, image contrast, small structure visibility, image sharpness, and artifacts. As radiation dose and strength level increased, image noise significantly decreased, CNR obviously increased, whilst CT value showed no significant difference (*P*>0.05). Results indicated that DLIR had better overall image quality than ASiR-V50%. Our finding was in accordance with Singh et al. (28) and Kim et al. (29)’s study that both obtained with relatively small sample sizes, but revealed a better significance due to the large patient cohort. Several studies suggested that DLIR was scored significantly better in overall image quality than different strengths of ASiR-V (level: 30%, 40%, and 50%) (24, 29) and comparable to ASiR-V (level: 70%, 100%) (30, 31).

Three HA inserts of 50.2-199.2 mg/cm^3^ provided a range of trabecular BMD mimicking the physiological range of BMD seen in all age groups (32). The relative measurement error of L1, L2, and L3 was 9.84%, 4.08%, and 2.60%, respectively. Coefficients of variance for the L1, L2, and L3 HA inserts were 1.51%, 1.41%, and 1.18%. Those all falling within the range of 4-15% and meeting the clinical BMD measurement requirements (4, 32, 33). The largest and smallest deviations were found in L3 and L1, respectively. As the BMD value decreased, the relative measurement error increased significantly; especially with BMD less than 100.2 mg/cm^3^, thus more attention should be paid to osteoporosis patients when evaluating the risk of osteoporotic fractures. Wu et al. (4) investigated the repeatability and accuracy of QCT measurement of BMD by low-mAs with iterative model reconstruction (IMR) algorithm based on phantom level and showed the maximum deviation of accuracy was 11% for L1, 4% for L2, and 6% for L3. In contrast, our study demonstrated that the accuracy of BMD at L1 and L3 was improved with DLIR in comparison to IMR (2), indicating that DLIR may potentially improve the low-contrast detectability and maintain the high-contrast spatial resolution. However, further studies should be implemented to verify whether DLIR can makes the images more homogeneous in terms of CT numbers. Consistent with our findings, Wang et al. (6) observed an excellent accuracy with 3 HA inserts ranging from 3.7% to 5.9%. Zhao et al. (16) found that the mean trabecular BMD measurement of 3 HA inserts were 2.4%, 2.1%, and 0.5% at L1, L2, and L3 for forty different systems on ESP, indicating a smaller measurement error than our study.

For patients aged over 50 years, the prevalence rate of osteoporosis was 24.67% in women and 11.79% in men, and it was comparable to 29.1% in women but more than twice in men by DXA, and similar to 29.0% in women and 13.5% in men by QCT reported by Cheng et al. (7). The prevalence rate of osteoporotic fracture was 8.67% in women and 5.29% in men, which was significantly lower than 17.3% in women and 17% in men for more than 14000 subjects in Shanghai conducted by Gao et al. (34). Conversely, a study in Norway enrolled 2887 participants demonstrated a higher prevalence rate of vertebral fracture 11.8% in women and 13.8% in men (35). The difference in osteoporotic fracture between DXA and QCT may be attributed to the patient cohort mostly obtained from the health check-up participants for osteoporosis screening, thus further studies should be performed to assess the fracture risk of QCT in multiple participants.

There are some limitations to be highlighted. Firstly, the results acquired with QCT should be further compared with DXA corresponding to the prevalence of osteoporosis. Secondly, a longitudinal study should be further performed to verify the clinical utility of DLIR algorithms in osteoporosis screening. Thirdly, we didn’t evaluate the risk factors of osteoporosis, such as age, BMI, smoking, and fragility fracture history.

In conclusion, image quality with DLIR was high-qualified without affecting the accuracy of BMD measurement. It may provide a great clinical utility in osteoporosis screening.

## Data availability statement

The raw data supporting the conclusions of this article will be made available by the authors, without undue reservation.

## Ethics statement

The studies involving human participants were reviewed and approved by the Ethics Committees of First Affiliated Hospital of Zhengzhou University. The patients/participants provided their written informed consent to participate in this study. Written informed consent was obtained from the individual(s) for the publication of any potentially identifiable images or data included in this article.

## Author contributions

YL, YJ, and YW designed the study. YL and YJ performed the data analysis. YL researched the related literatures. All authors contributed the data collection, measurements, and interpretation. YL wrote the manuscript and all authors reviewed the manuscript.

## Funding

This study is supported by the National Natural Science Foundation of China (grant no. U1504821).

## Conflict of interest

The authors declare that the research was conducted in the absence of any commercial or financial relationships that could be construed as a potential conflict of interest.

## Publisher’s note

All claims expressed in this article are solely those of the authors and do not necessarily represent those of their affiliated organizations, or those of the publisher, the editors and the reviewers. Any product that may be evaluated in this article, or claim that may be made by its manufacturer, is not guaranteed or endorsed by the publisher.

## References

[1] Liang C Zhang G Wang J Tang X Chen H Yu R. An epidemiological investigation of patients with hip fracture over 50 years old in changning district. Chin J Bone Joint Injury (2013) 28:1122–4. doi: 10.7531/j.issn.1672-9935.2013.12.004

[2] Nevitt MC Ross PD Palermo L Musliner T Genant HK Thompson DE. Association of prevalent vertebral fractures, bone density, and alendronate treatment with incident vertebral fractures: Effect of number and spinal location of fractures. Fract Intervent Trial Res Group Bone (1999) 25(5):613–9. doi: 10.1016/s8756-3282(99)00202-1 10574584

[3] Patil S Rawall S Singh D Mohan K Nagad P Shial B. Surgical patterns in osteoporotic vertebral compression fractures. Eur Spine J (2013) 22(4):883–91. doi: 10.1007/s00586-012-2508-4 PMC3631027 23053751

[4] Wu Y Guo Z Fu X Wu J Gao J Zeng Q. The study protocol for the China health big data (China biobank) project. Quant Imaging Med Surg (2019) 9(6):1095–102. doi: 10.21037/qims.2019.06.16 PMC6629571 31367563

[5] Wu Y Jiang Y Han X Wang M Gao J. Application of low-tube current with iterative model reconstruction on philips brilliance iCT elite FHD in the accuracy of spinal QCT using a European spine phantom. Quant Imaging Med Surg (2018) 8(1):32–8. doi: 10.21037/qims.2018.02.03 PMC5835662 29541621

[6] Wang L Su Y Wang Q Duanmu Y Yang M Yi C. Validation of asynchronous quantitative bone densitometry of the spine: Accuracy, short-term reproducibility, and a comparison with conventional quantitative computed tomography. Sci Rep (2017) 7(1):6284. doi: 10.1038/s41598-017-06608-y 28740145 PMC5524691

[7] Cheng X Zhao K Zha X Du X Li Y Chen S. China Health big data (China biobank) project investigators. opportunistic screening using low-dose CT and the prevalence of osteoporosis in China: A nationwide, multicenter study. J Bone Miner Res (2021) 36(3):427–35. doi: 10.1002/jbmr.4187 PMC7988599 33145809

[8] Cao L Liu X Li J Qu T Chen L Cheng Y. A study of using a deep learning image reconstruction to improve the image quality of extremely low-dose contrast-enhanced abdominal CT for patients with hepatic lesions. Br J Radiol (2021) 94(1118):20201086. doi: 10.1259/bjr.20201086 33242256 PMC7934287

[9] Han WK Na JC Park SY. Low-dose CT angiography using ASiR-V for potential living renal donors: a prospective analysis of image quality and diagnostic accuracy. Eur Radiol (2020) 30(2):798–805. doi: 10.1007/s00330-019-06423-1 31471753

[10] Liu L. Model-based iterative reconstruction: A promising algorithm for today's computed tomography imaging. J Med Imaging Radiat Sci (2014) 45(2):131–6. doi: 10.1016/j.jmir.2014.02.002 31051943

[11] Verdun FR Racine D Ott JG Tapiovaara MJ Toroi P Bochud FO. Image quality in CT: From physical measurements to model observers. Phys Med (2015) 31(8):823–43. doi: 10.1016/j.ejmp.2015.08.007 26459319

[12] Samei E Richard S. Assessment of the dose reduction potential of a model-based iterative reconstruction algorithm using a task-based performance metrology. Med Phys (2015) 42(1):314–23. doi: 10.1118/1.4903899 25563271

[13] JHsieh J Liu E Nett B Tang J Thibault JB Sahney S. A new era of image reconstruction: TrueFidelity technical white paper on deep learning image reconstruction. GE Healthcare (2019).

[14] Li Y Jiang Y Liu H Yu X Chen S Ma D. A phantom study comparing low-dose CT physical image quality from five different CT scanners. Quant Imaging Med Surg (2022) 12(1):766–80. doi: 10.21037/qims-21-245 PMC8666789 34993117

[15] Goodenough DJ. Catphan 500 and 600 manual. Greenwish, NY: The Phantom Laboratory, Inc (2006).

[16] Zhao Y Li K Duanmu Y Wang L Xu X Zhang Y. Accuracy, linearity and precision of spine QCT vBMD phantom measurements for different brands of CT scanner: A multicentre study. J Clin Densitom (2022) 25(1):34–42. doi: 10.1016/j.jocd.2021.02.004 33745832

[17] Abdullah KA McEntee MF Reed WM Kench PL. Increasing iterative reconstruction strength at low tube voltage in coronary CT angiography protocols using 3D-printed and catphan 500 phantoms. J Appl Clin Med Phys (2020) 21(9):209–14. doi: 10.1002/acm2.12977 PMC7497920 32657493

[18] Ehman EC Yu L Manduca A Hara AK Shiung MM Jondal D. Methods for clinical evaluation of noise reduction techniques in abdominopelvic CT. Radiographics (2014) 34(4):849–62. doi: 10.1148/rg.344135128 25019428

[19] Bujila R Kull L Danielsson M Andersson J. Applying three different methods of measuring CTDIfree air to the extended CTDI formalism for wide-beam scanners (IEC 60601-2-44): A comparative study. J Appl Clin Med Phys (2018) 19(4):281–9. doi: 10.1002/acm2.12363 PMC6036408 29900670

[20] Gulliksrud K Stokke C Martinsen AC. How to measure CT image quality: variations in CT-numbers, uniformity and low contrast resolution for a CT quality assurance phantom. Phys Med (2014) 30(4):521–6. doi: 10.1016/j.ejmp.2014.01.006 24530005

[21] PROTM QCT. Bone mineral density software. User’s Guide Mindways Soft Inc (2013).

[22] van Hamersvelt RW Schilham AMR Engelke K den Harder AM de Keizer B Verhaar HJ. Accuracy of bone mineral density quantification using dual-layer spectral detector CT: A phantom study. Eur Radiol (2017) 27(10):4351–9. doi: 10.1007/s00330-017-4801-4 PMC5579207 28374079

[23] Wong JC Griffiths MR. Precision of bone densitometry measurements: When is change true change and does it vary across bone density values? Australas Radiol (2003) 47(3):236–9. doi: 10.1046/j.1440-1673.2003.01169.x 12890241

[24] Nam JG Hong JH Kim DS Oh J Goo JM. Deep learning reconstruction for contrast-enhanced CT of the upper abdomen: Similar image quality with lower radiation dose in direct comparison with iterative reconstruction. Eur Radiol (2021) 31(8):5533–43. doi: 10.1007/s00330-021-07712-4 33555354

[25] Li G Gao G Xia H. Detection and influencing factors of CT spatial resolution and low-contrast resolution. China Med Dev (2010) 25:7–9. doi: 10.3969/j.issn.1674-1633.2010.01.003

[26] Greffier J Hamard A Pereira F Barrau C Pasquier H Beregi JP. Image quality and dose reduction opportunity of deep learning image reconstruction algorithm for CT: a phantom study. Eur Radiol (2020) 30(7):3951–9. doi: 10.1007/s00330-020-06724-w 32100091

[27] Higaki T Nakamura Y Zhou J Yu Z Nemoto T Tatsugami F. Deep learning reconstruction at CT: Phantom study of the image characteristics. Acad Radiol (2020) 27(1):82–7. doi: 10.1016/j.acra.2019.09.008 31818389

[28] Singh R Digumarthy SR Muse VV Kambadakone AR Blake MA Tabari A. Image quality and lesion detection on deep learning reconstruction and iterative reconstruction of submillisievert chest and abdominal CT. AJR Am J Roentgenol (2020) 214(3):566–73. doi: 10.2214/AJR.19.21809 31967501

[29] Kim JH Yoon HJ Lee E Kim I Cha YK Bak SH. Validation of deep-learning image reconstruction for low-dose chest computed tomography scan: Emphasis on image quality and noise. Kor J Radiol (2021) 22(1):131–8. doi: 10.3348/kjr.2020.0116 PMC7772377 32729277

[30] Sun J Li H Li J Yu T Li M Zhou Z. Improving the image quality of pediatric chest CT angiography with low radiation dose and contrast volume using deep learning image reconstruction. Quant Imaging Med Surg (2021) 11(7):3051–8. doi: 10.21037/qims-20-1158 PMC8250028 34249634

[31] Benz DC Benetos G Rampidis G von Felten E Bakula A Sustar A. Validation of deep-learning image reconstruction for coronary computed tomography angiography: Impact on noise, image quality and diagnostic accuracy. J Cardiovasc Comput Tomogr (2020) 14(5):444–51. doi: 10.1016/j.jcct.2020.01.002 31974008

[32] Kalender WA Felsenberg D Genant HK Fischer M Dequeker J Reeve J. The European spine phantom–a tool for standardization and quality control in spinal bone mineral measurements by DXA and QCT. Eur J Radiol (1995) 20(2):83–92. doi: 10.1016/0720-048x(95)00631-y 7588873

[33] Glüer CC Engelke K Lang TF Grampp S Genant HK. Quantitative computed tomography (QCT) of the lumbar spine and appendicular skeleton. Eur J Radiol (1995) 20(3):173–8. doi: 10.1016/0720-048x(95)00651-6 8536743

[34] Gao C Xu Y Li L Gu W Yi C Zhu Q. Prevalence of osteoporotic vertebral fracture among community-dwelling elderly in shanghai. Chin Med J (Engl) (2019) 132(14):1749–51. doi: 10.1097/CM9.0000000000000332 PMC6759100 31261209

[35] Waterloo S Ahmed LA Center JR Eisman JA Morseth B Nguyen ND. Prevalence of vertebral fractures in women and men in the population-based tromsø study. BMC Musculoskelet Disord (2012) 13:3. doi: 10.1186/1471-2474-13-3 22251875 PMC3273434

